# Genome sequence of *Cysteiniphilum* sp. GTC20157 isolated from coastal seawater in Osaka, Japan

**DOI:** 10.1128/mra.01243-25

**Published:** 2025-12-10

**Authors:** Masahiro Hayashi, Yushi Naka, Hiromi Machida, Masanori Miyata, Jun Yonetamari, Yoshinori Muto, Motoki Takagi, Kaori Tanaka

**Affiliations:** 1 Institute for Glyco-core Research iGCORE, Gifu University, Gifu City, Gifu, Japan; 2 Division of Anaerobe Research, Life Science Research Center, Gifu University 12785, Gifu City, Gifu, Japan; 3 Gifu University Center for Conservation of Microbial Genetic Resource, Gifu, Japan; 4 MIZUKEN Co., Ltd., Sakai City, Osaka, Japan; 5 Division of Clinical Laboratory, Gifu University Hospital 476117, Gifu, Japan; 6 United Graduate School of Drug Discovery and Medical Information Sciences, Gifu University 12785, Gifu City, Gifu, Japan; 7 JeiserBio Inc., Yokohama City, Kanagawa, Japan; Montana State University, Bozeman, Montana, USA

**Keywords:** *Cysteiniphilum*, genomes

## Abstract

We report the complete genome sequence of *Cysteiniphilum* strain GTC20157 isolated from coastal seawater in Osaka, Japan. The genome comprised a circular chromosome and a circular plasmid measuring 2,508,673 and 149,142 bp, respectively.

## ANNOUNCEMENT

*Cysteiniphilum* species belong to the family *Fastidiosibacteraceae* and are characterized as Gram-stain-negative, rod-shaped, non-motile, and aerobic bacteria. *Cysteiniphilum* species are commonly isolated from coastal environments (1–3). In this study, strain GTC20157 was isolated from coastal seawater collected in Osaka Prefecture, Japan. Seawater sample (500 mL) was collected from coastal seawater and concentrated 50 times by centrifugation at 1,200 × *g* for 20 min. From this concentrated sample, 0.2 mL was inoculated into buffered charcoal yeast extract (BCYE) agar and cultured at 37°C for 48 h under aerobic conditions. The isolate was cultured and purified twice under conditions mentioned above.

The strain was cultured on BCYE agar under the same conditions. Colonies were scraped, suspended in TE buffer, and pelleted via centrifugation. Genomic DNA was extracted using NucleoBond HMW DNA Kit (MACHEREY-NAGEL, Japan).

For identification, the *16S* rRNA gene was sequenced after PCR amplification using primers 16S-8F (AGAGTTTGATCMTGGCTCAG) and 16S-1492R (GGYTACCTTGTTACGACTT). BLASTN analysis (4) of the 16S rRNA gene sequence showed that strain GTC20157 was closely related to *Cysteiniphilum marinum* 19X3-30 (GenBank accession no. MW063583), *Cysteiniphilum halobium* SYSU SYW-6 (GenBank accession no. MH329654), and *Cysteiniphilum littorale* SYSU D3-2 (GenBank accession no. KX817994), with 98.5%, 96.6%, and 98.2% sequence identity, respectively.

The genome was sequenced using PromethION 2i (Oxford Nanopore Technologies [ONT], Oxford, UK) for long-read sequencing and DNBSEQ (MGI Tech Co., Ltd., Shenzhen, China) for short-read sequencing (5–7). The same DNA extract was used for both platforms. A library was constructed with a Native Barcoding Kit (SQK-NBD114-24; ONT) without shearing for long-read sequencing. Small DNA fragments were removed using the Short Read Eliminator XS Kit (Pacific Biosciences of California, Inc., USA). Sequencing was performed on a PromethION 2i (ONT) with a FLO-PRO114M flow cell. Base calling was performed with Dorado version 7.2.14 (ultra-high-accuracy mode), and raw reads were trimmed and quality-filtered using NanoFilt version 2.7.1 (8) with parameters “-l 1000 -q 10 --headcrop 50.” For short reads, the library was constructed using the MGIEasy FS DNA Library Prep Set (MGI Tech), and 2 × 150-bp paired-end sequencing was performed on the DNBSEQ-G400 platform (MGI Tech). Reads were processed with fastp version 0.20.1 (9) using “-q 30 -n 20 -t 1 -T 1.” Short-read quality was evaluated using fastp (9), and long-read quality was assessed using NanoPlot version 1.32.1 (9). Short reads (with over 94.5% of bases >Q30) and long reads (mean read quality of 18.8) were assembled using Unicycler version 0.4.8 (10) with default settings. Assembly circularization and rotation were performed automatically in a Unicycler. The contig graph was visualized with Bandage version 0.8.1 (11), and the taxonomic integrity was confirmed using Blobtools version 1.0 with short reads (12). Genome annotation was performed using DDBJ Fast Annotation and Submission Tool (DFAST) (13). The assembly was rotated to start with the *dnaA* gene on the forward strand.

Table 1 summarizes the genomic information. Unicycler and Bandage indicated a complete circular genome. Quality and genome statistics were computed using CheckM (version 1.2.2) (14) and seqkit (version 2.9.0) (15). CheckM confirmed that the genome was 99.6% complete with 0.2% contamination. DFAST (13) predicted 2,376 coding sequences, 12 rRNAs, 46 tRNAs, and 1 CRISPR sequence.

**TABLE 1:** Information on the complete genome sequence of a *Cysteiniphilum* sp. strain isolated from coastal seawater in Japan

	Strain name
Parameter	*Cysteiniphilum* sp*.* GTC20157
DNBSEQ sequencing^*a*^	
No. of reads	19,614,364
Size (kb)	2,942,155
Avg coverage (×)	1,172.70
DRA accession no.	DRR751259
ONT sequencing^*a*^	
No. of reads	303,863
Size (kb)	2,104,353.60
Avg read length (bp)	6,925.30
Avg coverage (×)	838.8
*N*_50_	59,121
DRA accession no.	DRR751260
Assembly	
Assembly *N*_50_ (bp)^*c*^	2,508,673
Estimated genome completeness (%)^*d*^	99.6
Estimated genome contamination (%)^*d*^	0.2
Genome structure	One chromosome and one plasmid
DDBJ/GenBank accession no.	AP044698 (GTC20157) AP044699 (pGTC20157)
Genome size (bp)	2,508,673 (GTC20157) 149,142 (pGTC20157)
GC content (%)	
(Chromosome/plasmid name)	38.7 (GTC20157) 36.8 (pGTC20157)
No. of coding sequences^*b*^	2,376
Number of rRNAs^*b*^	12
Number of tRNAs^*b*^	46
Number of CRISPRs^*b*^	1

^*a*^ DRA, DDBJ Sequence Read Archive.

^*b*^ DFAST, DDBJ Fast Annotation and Submission Tool.

^*c*^ Determined with seqkit version 2.3.0.

^*d*^ Determined with CheckM version 1.2.2.

## Data Availability

The genome sequence of *Cysteiniphilum* sp. (GTC20157) has been deposited into DDBJ (https://www.ddbj.nig.ac.jp/index.html) under the accession numbers AP044698 and AP044699. Raw sequence data for GTC20157 have been deposited into the DDBJ/Sequence Read Archive under accession numbers DRR751259 and DRR751260.
